# Notification that new names of prokaryotes, new combinations and new taxonomic opinions have appeared in volume 76, part 4 of the IJSEM

**DOI:** 10.1099/ijsem.0.007198

**Published:** 2026-07-31

**Authors:** Aharon Oren, Markus Göker

**Affiliations:** 1 The Institute of Life Sciences, The Hebrew University of Jerusalem, The Edmond J. Safra Campus, 9190401 Jerusalem, Israel; 2 Leibniz Institute DSMZ – German Collection of Microorganisms and Cell Cultures, Inhoffenstrasse 7B, 38124 Braunschweig, Germany

**Keywords:** list, names, notification

This listing of names of prokaryotes published in a previous issue of the *International Journal of Systematic and Evolutionary Microbiology* (*IJSEM*) is provided as a service to microbiology to assist in the recognition of new names and new combinations. This procedure was proposed by the Judicial Commission [1]. The names given herein are listed according to the rules of priority (i.e. the time and date of publication of the articles on the journal’s platform and the order of valid publication of names in the original articles) [2].

**Table:** 

Order of article publication	Name/authors	Proposed as	Article no.	Page no.
1	*Qipengyuania triglochinis* Yamamoto *et al*. 2026	sp. nov.	007113	9
1	*Alteriqipengyuania triglochinis* Yamamoto *et al*. 2026	sp. nov.	007113	9
2	*Streptomyces dryopteridis* Gao *et al*. 2026	sp. nov.	007104	8
2	*Streptomyces sparsogenes* subsp. *sparsogenes* (Owen *et al*. 1963) Gao *et al*. 2026	comb. nov. [basonym: *Streptomyces sparsogene*s Owen *et al*. 1963 (Approved Lists 1980)]	007104	9
2	*Streptomyces sparsogenes* subsp. *cuspidosporus* (Higashide *et al*. 1966) Gao *et al*. 2026	comb. nov. [basonym: *Streptomyces cuspidosporus* Higashide *et al*. 1966 (Approved Lists 1980)]	007104	10
3	*Prevotella mikamonis* Hayashi *et al*. 2026	sp. nov.	007112	8
4	*Parvimonas oralis* Gao *et al*. 2026	sp. nov.	007123	6
4	*Parvimonas pharyngis* Gao *et al*. 2026	sp. nov.	007123	7
5	*Gordonia abscondita* Guzman *et al*. 2026	sp. nov.	007114	10
5	*Pseudochrobactrum mosebachae* Guzman *et al*. 2026	sp. nov.	007114	11
6	*Paenibacillus hebaui* Yan *et al*. 2026	sp. nov.	007120	8
7	*Phreatobacter aquiterrae* Cozannet *et al*. 2026	sp. nov.	007109	10
8	*Paenibacillus oryziterrae* Liu *et al*. 2026	sp. nov.	007124	14
8	*Paenibacillus paludis* Liu *et al*. 2026	sp. nov.	007124	15
8	*Paenibacillus diazotrophicus* Liu *et al*. 2026	sp. nov.	007124	15
9	*Bradyrhizobium cedriense* Jihnaoui *et al*. 2026	sp. nov.	007127	10
10	*Terasakiella magnetica* Koziaeva *et al*. 2026	sp. nov.	007129	7
10	*Magnetovibrio massiliensis* Koziaeva *et al*. 2026	sp. nov.	007129	8
10	*Magnetococcus organivorans* Koziaeva *et al*. 2026	sp. nov.	007129	8
11	*Microbacterium songi* Pu *et al*. 2026	sp. nov.	007116	10
11	*Microbacterium hanjuni* Pu *et al*. 2026	sp. nov.	007116	10
11	*Microbacterium shimangi* Pu *et al*. 2026	sp. nov.	007116	10
12	*Allohoeflea* Naranjo-Robayo *et al*. 2026	gen. nov.	007131	13
12	*Arminella* Naranjo-Robayo *et al*. 2026	gen. nov.	007131	13
12	*Gillisella* Naranjo-Robayo *et al*. 2026	gen. nov.	007131	13
12	*Limnomicrobium* Naranjo-Robayo *et al*. 2026	gen. nov.	007131	13
12	*Martinezella* Naranjo-Robayo *et al*. 2026	gen. nov.	007131	13
12	*Neohoeflea* Naranjo-Robayo *et al*. 2026	gen. nov.	007131	13
12	*Parahoeflea* Naranjo-Robayo *et al*. 2026	gen. nov.	007131	14
12	*Velazquezella* Naranjo-Robayo *et al*. 2026	gen. nov.	007131	14
12	*Affinirhizobium capsici* (Lin *et al*. 2015) Naranjo-Robayo *et al*. 2026	comb. nov. [basonym: *Rhizobium capsici* Lin *et al*. 2015]	007131	14
12	*Affinirhizobium straminoryzae* (Lin *et al*. 2014) Naranjo-Robayo *et al*. 2026	comb. nov. [basonym: *Rhizobium straminoryzae* Lin *et al*. 2014]	007131	14
12	*Aliirhizobium zeae* (Celador-Lera *et al*. 2017) Naranjo-Robayo *et al*. 2026	comb. nov. [basonym: *Rhizobium zeae* Celador-Lera *et al*. 2017]	007131	14
12	*Allohoeflea poritis* (Zhang *et al*. 2023) Naranjo-Robayo *et al*. 2026	comb. nov. [basonym: *Hoeflea poritis* Zhang *et al*. 2023]	007131	14
12	*Arminella rhododendri* (Kuzmanović *et al*. 2023) Naranjo-Robayo *et al*. 2026	comb. nov. [basonym: *Rhizobium rhododendri* Kuzmanović *et al*. 2023]	007131	15
12	*Arminella tubonensis* (Zhang *et al*. 2011) Naranjo-Robayo *et al*. 2026	comb. nov. [basonym: *Rhizobium tubonense* Zhang *et al*. 2011]	007131	15
12	*Arminella tumorigenes* (Kuzmanović *et al*. 2019) Naranjo-Robayo *et al*. 2026	comb. nov. [basonym: *Rhizobium tumorigenes* Kuzmanović *et al*. 2019]	007131	15
12	*Gillisella ampelina* (Kuzmanović *et al*. 2022) Naranjo-Robayo *et al*. 2026	comb. nov. [basonym: *Allorhizobium ampelinum* Kuzmanović *et al*. 2022]	007131	15
12	*Gillisella taibaishanensis* (Yao *et al*. 2012) Naranjo-Robayo *et al*. 2026	comb. nov. [basonym: *Rhizobium taibaishanense* Yao *et al*. 2012]	007131	15
12	*Gillisella terrae* (Lin *et al*. 2020) Naranjo-Robayo *et al*. 2026	comb. nov. [basonym: *Allorhizobium terrae* Lin *et al*. 2020]	007131	16
12	*Gillisella vitis* (Ophel and Kerr 1990) Naranjo-Robayo *et al*. 2026	comb. nov. [basonym: *Agrobacterium vitis* Ophel and Kerr 1990]	007131	16
12	*Limnomicrobium aquaticum* (Máthé *et al*. 2019) Naranjo-Robayo *et al*. 2026	comb. nov. [basonym: *Rhizobium aquaticum* Máthé *et al*. 2019]	007131	16
12	*Martinezella calliandrae* (Rincón-Rosales *et al*. 2013) Naranjo-Robayo *et al*. 2026	comb. nov. [basonym: *Rhizobium calliandrae* Rincón-Rosales *et al*. 2013]	007131	16
12	*Martinezella dioscoreae* (Ouyabe *et al*. 2020) Naranjo-Robayo *et al*. 2026	comb. nov. [basonym: *Rhizobium dioscoreae* Ouyabe *et al*. 2020]	007131	16
12	*Martinezella freirei* (Dall’Agnol *et al*. 2013) Naranjo-Robayo *et al*. 2026	comb. nov. [basonym: *Rhizobium freirei* Dall’Agnol *et al*. 2013]	007131	17
12	*Martinezella hainanensis* (Chen *et al*. 1997) Naranjo-Robayo *et al*. 2026	comb. nov. [basonym: *Rhizobium hainanense* Chen *et al*. 1997]	007131	17
12	*Martinezella jaguaris* (Rincón-Rosales *et al*. 2013) Naranjo-Robayo *et al*. 2026	comb. nov. [basonym: *Rhizobium jaguaris* Rincón-Rosales *et al*. 2013]	007131	17
12	*Martinezella leucaenae* (Ribeiro *et al*. 2012) Naranjo-Robayo *et al*. 2026	comb. nov. [basonym: *Rhizobium leucaenae* Ribeiro *et al*. 2012]	007131	17
12	*Martinezella lusitana* (Valverde *et al*. 2006) Naranjo-Robayo *et al*. 2026	comb. nov. [basonym: *Rhizobium lusitanum* Valverde *et al*. 2006]	007131	17
12	*Martinezella multihospitum* corrig. (Han *et al*. 2008) Naranjo-Robayo *et al*. 2026	comb. nov. [basonym: *Rhizobium multihospitium* Han *et al*. 2008]^†^	007131	18
12	*Martinezella miluonensis* (Gu *et al*. 2008) Naranjo-Robayo *et al*. 2026	comb. nov. [basonym: *Rhizobium miluonense* Gu *et al*. 2008]	007131	18
12	*Martinezella paranaensis* (Dall’Agnol *et al*. 2014) Naranjo-Robayo *et al*. 2026	comb. nov. [basonym: *Rhizobium paranaense* Dall’Agnol *et al*. 2014]	007131	18
12	*Martinezella rhizogenes* (Riker *et al*. 1930) Naranjo-Robayo *et al*. 2026	comb. nov. [basonym: ‘*Phytomonas rhizogenes*’ Riker *et al*. 1930]^‡^	007131	18
12	*Martinezella tropici* (Martínez-Romero *et al*. 1991) Naranjo-Robayo *et al*. 2026	comb. nov. [basonym: *Rhizobium tropici* Martínez-Romero *et al*. 1991]	007131	18
12	*Neohoeflea coraliihabitans* (Yu *et al*. 2024) Naranjo-Robayo *et al*. 2026	comb. nov. [basonym: *Pseudohoeflea coraliihabitans* Yu *et al*. 2024]	007131	19
12	*Neorhizobium puerariae* (Boonsnongcheep *et al*. 2016) Naranjo-Robayo *et al*. 2026	comb. nov. [basonym: *Rhizobium puerariae* Boonsnongcheep *et al*. 2016]	007131	19
12	*Parahoeflea alexandrii* (Palacios *et al*. 2006) Naranjo-Robayo *et al*. 2026	comb. nov. [basonym: *Hoeflea alexandrii* Palacios *et al*. 2006]	007131	19
12	*Parahoeflea halophila* (Jung *et al*. 2013) Naranjo-Robayo *et al*. 2026	comb. nov. [basonym: *Hoeflea halophila* Jung *et al*. 2013]	007131	19
12	*Parahoeflea olei* (Rahul *et al*. 2015) Naranjo-Robayo *et al*. 2026	comb. nov. [basonym: *Hoeflea olei* Rahul *et al*. 2015]	007131	19
12	*Parahoeflea phototrophica* (Biebl *et al*. 2006) Naranjo-Robayo *et al*. 2026	comb. nov. [basonym: *Hoeflea phototrophica* Biebl *et al*. 2006]	007131	20
12	*Sinorhizobium aridi* (Rocha *et al*. 2024) Naranjo-Robayo *et al*. 2026	comb. nov. [basonym: *Ensifer aridi* Rocha *et al*. 2024]	007131	20
12	*Velazquezella soli* (Yoon *et al*. 2010) Naranjo-Robayo *et al*. 2026	comb. nov. [basonym: *Rhizobium soli* Yoon *et al*. 2010]	007131	20
13	*Sphingopyxis selenitireducens* Liu *et al*. 2026	sp. nov.	007126	10
13	*Paeniglutamicibacter selenitireducens* Liu *et al*. 2026	sp. nov.	007126	10
14	*Hydrophilobacter* Choi *et al*. 2026	gen. nov.	007122	9
14	*Hydrophilobacter chitinilyticus* Choi *et al*. 2026	sp. nov.	007122	9
15	*Thiemannella* Robertson and Meyers 2026	gen. nov.	007135	11
15	*Thiemannella venezuelensis* (Thiemann 1970) Robertson and Meyers 2026	comb. nov. [basonym: *Planomonospora venezuelensis* Thiemann 1970 (Approved Lists 1980)]	007135	11
15	*Paraplanobispora* Robertson and Meyers 2026	gen. nov.	007135	12
15	*Paraplanobispora siamensis* (Ngaemthao *et al*. 2013) Robertson and Meyers 2026	comb. nov. [basonym: *Planobispora siamensis* Ngaemthao *et al*. 2013]	007135	12
15	*Paraplanobispora takensis* (Ngaemthao *et al*. 2014) Robertson and Meyers 2026	comb. nov. [basonym: *Planobispora takensis* Ngaemthao *et al*. 2014]	007135	12
15	*Planomonospora antibiotica* (Thiemann *et al*. 1968) Robertson and Meyers 2026	comb. nov. [basonym: *Planomonospora parontospora* subsp. *antibiotica* Thiemann *et al*. 1968 (Approved Lists 1980)]	007135	12
15	*Planomonospora parontospora* subsp. *sphaerica* (Mertz 1994) Robertson and Meyers 2026	comb. nov. [basonym: *Planomonospora sphaerica* Mertz 1994]	007135	13
16	*Desulforhopalus glucosivorans* Watanabe *et al*. 2026	sp. nov.	007080	5
17	*Luteimonas campi* Xu *et al*. 2026	sp. nov.	007133	9
18	*Dehalogenimonas loeffleri* Wang *et al*. 2026	sp. nov.	007137	10
19	*Pseudomonas citrullilanati* Fullem *et al*. 2026*	nom. nov. [illegitimate basonym: *Pseudomonas citrulli* Fullem *et al*. 2024]	007147	1
20	*Methanobacterium nebraskense* Fiore *et al*. 2026	sp. nov.	007139	8
21	*Mycolicibacterium chengduense* Wu *et al*. 2026	sp. nov.	007138	9
21	*Mycolicibacterium sichuanense* Wu *et al*. 2026	sp. nov.	007138	9
22	*Staphylococcus caseorum* Vázquez *et al*. 2026	sp. nov.	007144	13
23	*Campylobacter parajejuni* Vu *et al*. 2026	sp. nov.	007151	9
24	*Chryseobacterium electrogenes* Yang *et al*. 2026	sp. nov.	007143	9
25	*Nocardioides asiaticus* Le and Kim 2026	sp. nov.	007146	8
25	*Nocardioides pollutisoli* Le and Kim 2026	sp. nov.	007146	8

*Replacement name for the illegitimate name *Pseudomonas citrulli* Fullem *et al*. 2024.

†The spelling of the epithet was corrected by the authors when forming the new combination. In line with Judicial Opinion 132 [3], the basonym should be corrected to* Rhizobium multihospitum* corrig. Han *et al*. 2008.

‡The authors erroneously gave *Rhizobium rhizogenes* (Riker *et al*. 1930) Young *et al*. 2001 as basonym.

The following taxonomic opinions (i.e. the emendation of circumscriptions or the creation of synonyms) do not affect the status of a name under the International Code of Nomenclature of Prokaryotes. These taxonomic opinions can neither be considered approved nor disapproved by the International Committee on Systematics of Prokaryotes.

**Table:** 

Name/authors:	Article no.	Page no.
*Magnetococcus* Bazylinski *et al*. 2013 emend. Koziaeva *et al*. 2026	007129	9
*Planomonospora* Thiemann *et al*. 1967 (Approved Lists 1980) emend. Robertson and Meyers 2026	007135	11
*Streptomyces cuspidosporus* Higashide *et al*. 1966 (Approved Lists 1980) pro synon. *Streptomyces sparsogenes* Owen *et al*. 1963 (Approved Lists 1980)	007104	8
*Streptomyces sparsogenes* Owen *et al*. 1963 (Approved Lists 1980) emend. Gao *et al*. 2026	007104	9
*Terasakiella* Satomi *et al*. 2002 emend. Koziaeva *et al*. 2026	007129	7

## Abbreviations

IJSEM: International Journal of Systematic and Evolutionary Microbiology
